# Overwhelming glycyrrhizin production in *Glycyrrhiza glabra* induced by rihizobial symbiosis

**DOI:** 10.1007/s11418-025-01918-2

**Published:** 2025-05-25

**Authors:** Shion Yamamoto, Aya Shimomura, Satoshi Watanabe, Mareshige Kojoma, Akihiro Suzuki

**Affiliations:** 1 The United Graduate School of Agricultural Sciences, Kagoshima University, 1-21-24 Korimoto, Kagoshima, 890-0065 Japan; 2 Faculty of Agriculture, Saga University, 1 Honjo-machi, Saga, 840-8502 Japan; 3 School of Pharmaceutical Sciences, Health Sciences University of Hokkaido, 1757 Kanazawa, Isgikari-gun, Hokkaido 061-0293 Japan

**Keywords:** *Glycyrrhiza*, Glycyrrhizic acid, Nodulation, Rhizobium, Symbiosis

## Abstract

**Abstract:**

We reported that *Glycyrrhiza uralensis* inoculated with rhizobium tended to increase biomass production and glycyrrhizic acid (GL) production, in this study we have also achieved drastically increase in biomass and GL production in *Glycyrrhiza glabra*. At thirty days after inoculation (DAI), a significant increase in SPAD values was observed, and the expression of GL synthesis marker genes was also significantly increased. At 150 DAI, a significant increase in biomass was observed. Characteristically, it was also found that thick roots were enlarged by rhizobial inoculation. In addition, the expression of GL synthesis marker genes was also significantly increased. Moreover, GL content per unit root dry weight reached 4%, and GL production per plant increased six times compared to uninoculated plants. Moreover, we tried to reveal the mechanism of induction of GL production by rhizobial inoculation. Since it has been reported that the expression of jasmonic acid (JA) synthesis marker genes is increased by rhizobium in soybean, we investigated the expression of those genes in *G. glabra*, and found that *GgMYC2* and *GgJAR1* were up-regulated at Thirty DAI. Furthermore, methyl jasmonate treatment increased the expression of GL synthesis marker genes, suggesting that JA signaling is involved in the increased GL production due to rhizobial inoculation. These results aid in understanding the mechanism of increased GL production through the introduction of rhizobial symbiosis, and show the potential for providing a technology to significantly shorten the cultivation period for the production of *Glycyrrhiza* that meets the criteria for herbal medicines.

**Graphical abstract:**

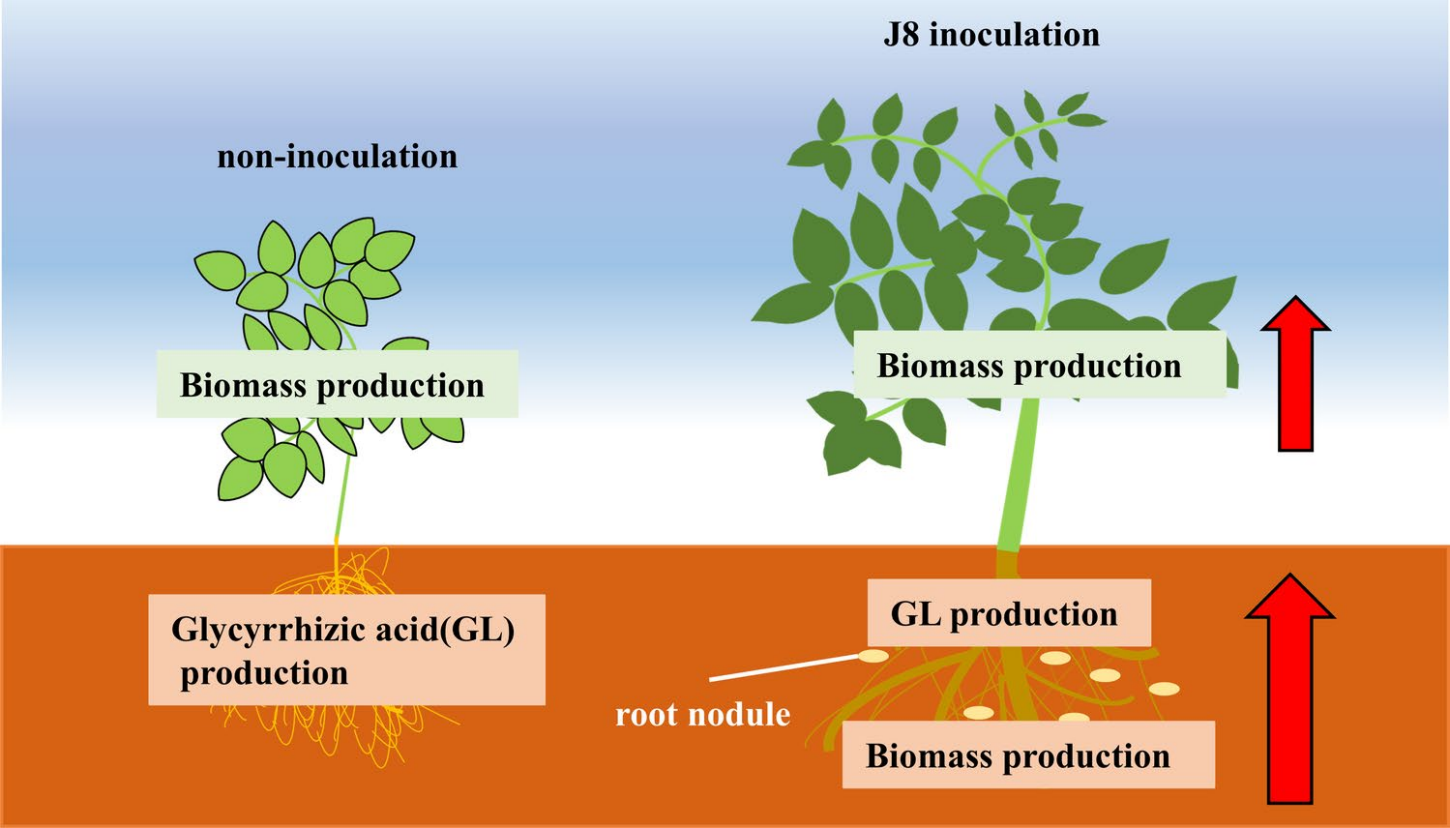

## Introduction

*Glycyrrhiza* is a medicinal plant of the legume family and is widely used as a crude drug in Kampo Medicine and Chinese-herbal medicines, as well as a natural sweetener. As a result, the size of the global market is growing year by year, with global trade value of approximately US$260 million in 2019 [1]. There are two types of *Glycyrrhiza* recognized as crude drugs in Japan: *Glycyrrhiza uralensis* and *Glycyrrhiza glabra*. While *G. uralensis* is used exclusively as a crude drug, *G. glabra* is in great demand for cosmetic additives, medicines, herbs, sweeteners, and other non-crude drugs [2]. However, these are not native to Japan, with *G. uralensis* growing wild in Central Asia, northern China, Mongolia, and other regions, and *G. glabra* growing wild in southern Europe and western China, and the *Glycyrrhiza* used in Japan is currently dependent on imports from China and Mongolia [3]. In recent years, desertification due to overharvesting of wild species has been occurring, making it difficult to supply good quality *Glycyrrhiza* [4], and the development of efficient domestic cultivation methods is required to ensure a stable supply of *Glycyrrhiza*.

Glycyrrhizic acid (GL, glycyrrhizin), a functional element, is contained in the roots and stolons of the plant, and it is stipulated that to be used as an herbal medicine, the plant must contain more than 2% of GL on a dry root and stolon weight basis (The Japanese Pharmacopoeia [5]). However, it takes more than three years to cultivate the plant to meet the standard using the current general cultivation methods. Therefore, in recent years, various studies have been conducted to develop efficient methods of growing plants that conform to standards. For example, tube cultivation using polyvinyl chloride pipes has been successful in efficiently extending roots vertically [6], but problems of cost and waste pipe disposal have been pointed out [7]. There is also a report of successful GL production by introducing the *CYP88D6* gene and the *UGT73P12* gene, which are involved in the biosynthetic pathway of GL, into yeast for efficient production of functional ingredients [8], but the production efficiency is low and this method cannot be used for crude drugs in the first place.

GL is a specialized metabolite, but because higher plants often produce specialized metabolites when they grow in excessively stressful environment [9–11], it is generally expected that plant biomass production will decrease in growth environments where GL production of *Glycyrrhiza* increases. In other words, a trade-off exists between biomass production and GL production. Therefore, to achieve compatibility between biomass production and GL production, it is necessary to resolve this trade-off. We have considered the use of rhizobial symbiosis in the cultivation of *Glycyrrhiza* to resolve this trade-off.

*Glycyrrhiza* is a perennial plant of the legume family. Leguminous plants such as *Glycine max*, *Lotus japonicus* can establish a symbiosis relationship with rhizobia, which form nodules on roots of the host plant and have the ability supply the plant with atmospheric nitrogen fixed in the nodules. Therefore, it is thought that *Glycyrrhiza* can also establish a symbiotic relationship with rhizobia. The supply of nitrogen sources to plants through rhizobial nitrogen fixation plays an extremely important role in legume biomass production. While rhizobial symbiosis promotes biomass production, rhizobium infection can also be a biotic stress for host plants [12]. Therefore, rhizobial symbiosis in *Glycyrrhiza* has the potential to resolve the trade-off between increased production of biomass and specialized metabolites. The nodulation on the roots of cultivated *Glycyrrhiza* is so rare that the impact of rhizobial symbiosis on GL production of *Glycyrrhiza* has not been investigated. Therefore, we isolated and identified a rhizobium (*Mesorhizobium* sp. J8) (strain J8) that efficiently establishes symbiosis with *G. uralensis*, and reported that inoculation with the rhizobium promotes GL production in 90 days inoculation experiment [13]. On the other hand, even though strain J8 was isolated from nodule on *G. glabra* roots, no nodulation on *G. glabra* was observed in re-inoculation experiments.

In this study, the inoculation conditions of strain J8 were reviewed to establish a symbiotic relationship between *G. glabra* and strain J8. In addition, the effects of strain J8 inoculation on the growth and GL production of *G. glabra* were investigated at different growth stages, 30 and 150 days after rhizobial inoculation.

## Materials and methods

### Soil materials

The soil used was a mixture of equal parts by volume of vermiculite, river sand, and sod soil (Green Sangyo). The vinyl pots (diameter 11 cm, height 24 cm) were filled with mixed soil.

### Plant materials

*G. glabra* (strain Hgl-1) was used in this experiment. Voucher specimens (accession nos.: *G. glabra*, GLY-GLA-001) were deposited in the Herbarium of the Faculty of Pharmaceutical Sciences, Health Sciences University of Hokkaido, Japan. The seedlings of *G. glabra* were prepared from cuttings. The cuttings were prepared by cutting shoots from the parent plants which were genetically identical at three leaves from the top. Basal ends of the shoots were cut horizontally and immediately immersed in water at 25 °C for 30 min. After immersion, the cuttings were inserted vertically into wet soil in vinyl pots. Water was sprayed on the leaves of the cuttings every day. The cuttings were supplied with tap water at their base for about a week after cutting and then with liquid B&D medium containing 1 mM KNO_3_. The plants were grown in liquid B&D medium containing 1 mM KNO_3_ for 3 weeks before being used for root nodulation tests.

### Preparation of rhizobium and rhizobial inoculation

The rhizobium used in this study was the same as those described in Kusaba et al. 2021 [13]. The culture of rhizobium, and inoculation to the plant also follow the method of Kusaba et al. 2021 [13]. Inoculations were performed on plants that had been nutritionally propagated by cuttings and rooted.

### Growth conditions of *G. glabra*

Strain J8 was diluted with liquid B&D medium [14] containing 1 mM KNO_3_ to 1.0 × 10^8^ cells/ml and 200 ml of it was added to the soil. As a control, liquid B&D medium containing 1 mM KNO_3_ was given. The surface of the soil was covered by cling film to prevent contamination. Other growth conditions were light/dark: 16 h/8 h, temperature: 25 °C and B&D liquid medium containing 1 mM KNO_3_ was applied once a week.

### Measurement of SPAD value

To investigate the chlorophyll content of leaves, the SPAD values of leaves were measured. The SPAD value was measured with a SPAD chlorophyll meter (SPAD-502Plus, Minolta Camera Co. Osaka, Japan). The average value of three leaves was used for each plant.

### Acetylene reduction assay (ARA) for nitrogenase activity

To investigate the nitrogen-fixing ability of symbiotic rhizobia, acetylene reduction activity of nitrogenase, an indicator of nitrogen-fixing activity, was measured [15]. The roots with nodule were placed in a 70-ml test tube or 600 ml flask and covered with a rubber cap. When test tubes were used, they were degassed for 1 min, and 5 times diluted acetylene was injected into the test tubes. When using a flask, 120 ml of air was removed from the flask using a 50 ml syringe (Terumo, Tokyo, Japan), and the same volume of pure acetylene was injected. After 1 h of incubation, the amount of ethylene was measured using a Shimadzu Gas Chromatograph [GC-14 (Shimadzu, Kyoto, Japan)].

### Measurements of root and stem diameter

The diameter was measured 1 cm above and below the base of the plant. For root diameter, three of the thickest adventitious roots that had rooted from the base were selected, and the average of the three was used as the root diameter.

### Expression analysis of GL and jasmonic acid (JA) synthesis marker genes

After growth measurements, the plant roots were quickly frozen in liquid nitrogen and stored at −80 °C. The preserved roots were finely crushed using a Multi-beads Shocker (Yasui Kikai, Japan). Total RNA was extracted using the RNeasy Plant Mini Kit (Qiagen, Basel, Switzerland). DNase I treatment was performed using RNase-Free DNase Set (Qiagen, Basel, Switzerland). Quantitative PCR was performed using the One Step TB Green PrimeScript PLUS RT-PCR Kit (Takara, Shiga, Japan) and the Applied Biosystems StepOne Real-Time PCR System (Thermo Fisher SCIENTIFIC, Massachusetts, America). Total RNA of plants 150 days after rhizobial inoculation was extracted from thick roots with a root diameter of at least 2 mm at 1 cm from the base. If thick roots were not present, the whole root was used for extraction. Gene-specific primer sequences were as follows: 18S ribosomal RNA (Accession No.:X02623) (5′-CTCAACACGGGG AAACTTAC-3′ and 5′-AGACAAATCGCTCCACCAAC-3′), *CYP88D6* (Accession No: MW071146) (5′-CGGAACATAGGCTTTTTCGAC-3′ and 5′-TACATTGCTAGCGCC TTGTG-3′), *CYP72A154* (Accession No.: MK534533) (5′-TGAAACTTGGAAACCTTTTGC-3′ and 5′-TCAAAAGTATTGGTACGGAAACC-3′), *CSyGT* (Accession No.: LC500232) (5′-TGAACACTCTGGTCTCTGCCCTTGC-3′ and 5′-CGAACTCAGAAGCCTCTCTCACGCC-3′), *JAR1* (5'-TCAAGGTGAGAGTGACCCTTC-3' and 5'-TTGACGGAATCTCTCGCTTC-3') and *MYC2* (5'-GAGGGTGCCAAGGGAGAG-3' and 5'-TCTTCACCAACCTTCACTTCAG-3'). The 18S ribosomal RNA gene was used as an internal control [16].

### Measurement of GL content

The GL content of *G. glabra* roots were measured by a modified method described by Kojoma et al. 2010 [17]. The HPLC system used consisted of a high-performance liquid chromatography (Chromaster 5310, Hitachi, Tokyo, Japan) and an InsertSustain C18, 5 μm HPLC column (GL Sciences Inc. Tokyo, Japan). Acetonitrile and formic acid were used as the solvents. The column oven was set to 40 °C, the column flow rate was set to 0.6 ml/min, and detection was performed at 240 to 260 nm. The samples used for HPLC were prepared as follows: 2 g of *G. glabra* root powder was added to 20 ml of 50% EtOH and sonicated for 30 min. After sonication, the samples were vortexed vigorously for 10 s and centrifuged (6000 rpm, 3 min) and the supernatant was filtered for analysis.

### Methyl jasmonate (MeJA) treatment

*G. glabra* plants without inoculation were grown for 30 days and given 200 ml of 10 µM or 100 µM MeJA solution. After 24 h, plant roots were collected and instantly frozen in liquid nitrogen.

## Results

### Confirmation of nodulation by inoculation of strain J8 in* G. glabra*

**Fig. 1:**
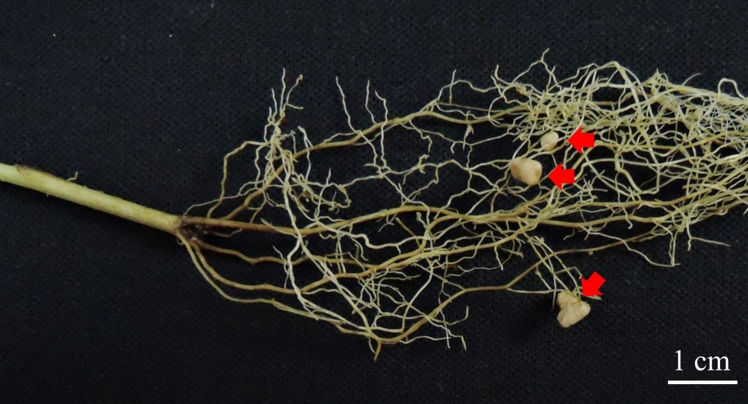
Phenotype of root nodule formed on the *G. glabra* prepared from cuttings and grown at 30 days after rhizobial inoculation. Arrows indicate nodules formed on the roots

In a previous study, we found that a rhizobium (*Mesorhizobium* sp. J8) promoted the growth of *G. uralensis* and increased the production of GL [13]. Although strain J8 was isolated from nodules formed on *G. glabra* roots, no nodules formed after 4 weeks in *G. glabra* that has been re-inoculated with strain J8 using nitrogen-free medium [13]. Therefore, in this study, we searched for conditions under which strain J8 forms nodules on *G. glabra.* Since nitrogen nutrition has been reported to be a positive regulator of nodulation in some plants [18, 19], 1 mM KNO_3_ was added to the medium to investigate nodulation by strain J8 inoculation. As a result, nodulation was observed in plants grown for 30 days (Fig. 1).

### Effect of strain J8 on the growth of *G. glabra* at 30 DAI.

**Fig. 2:**
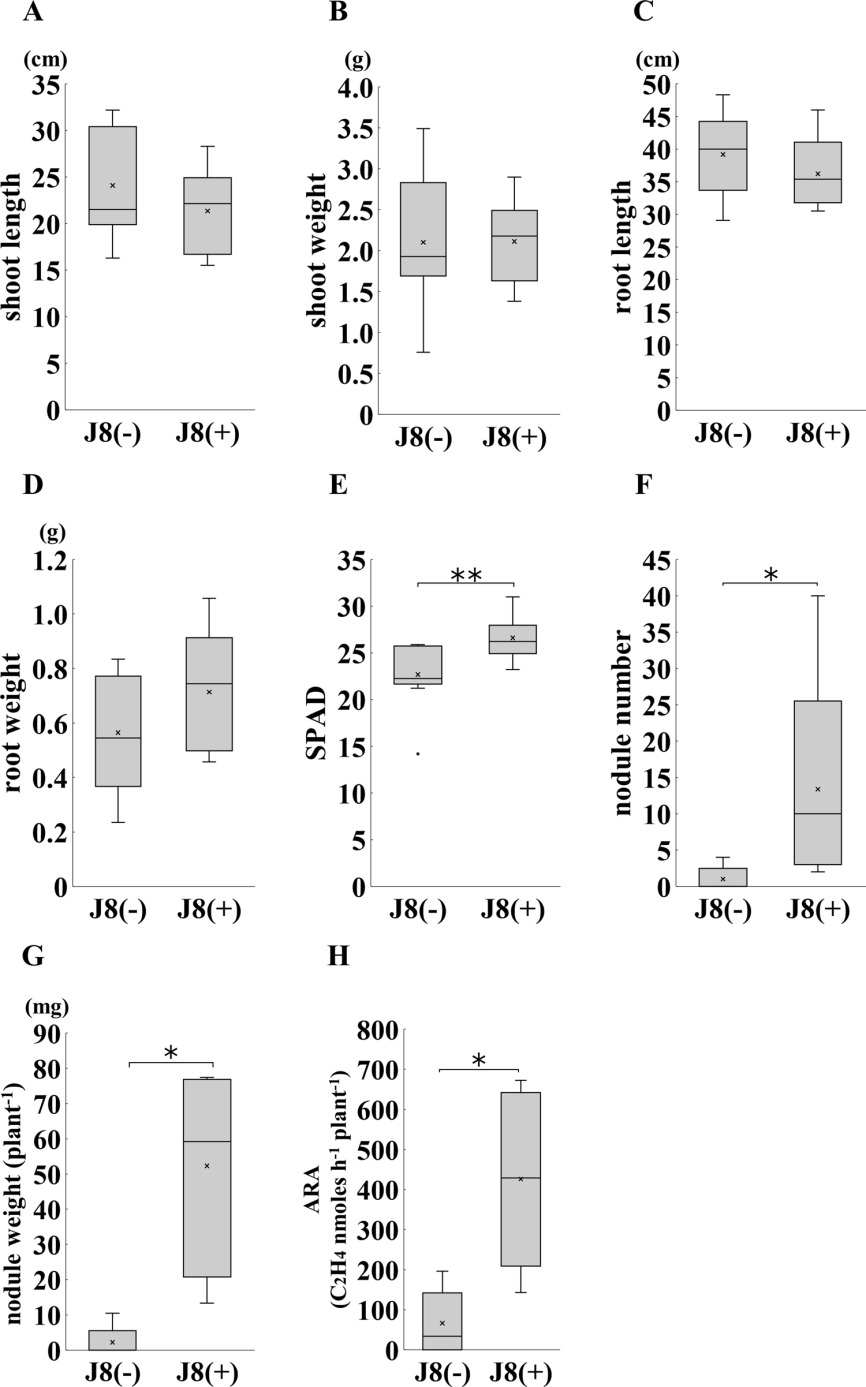
Growth, nodulation and nitrogen fixation ability of *G. glabra* prepared from cuttings at 30 days after rhizobial inoculation. J8(−) indicates uninoculated plants and J8(+) indicates plants inoculated with strain J8. Shoot length (**A**), shoot weight (**B**), root length (**C**), root weight (**D**) were measured. Leaf SPAD values (E) were measured on three leaves of each plant and the average value was calculated. Number of nodules (**F**), nodule weight (**G**), acetylene reduction activity (**H**) were measured. Shoot length and weight; J8(−), J8(+): *n* = 11, *n* = 10, respectively. Root length and weight; *n* = 5. Leaf SPAD value; *n* = 10. Nodule number and acetylene reduction activity; J8(−), J8(+):*n* = 7, *n* = 5, respectively. Nodule weight; J8(−), J8(+):*n* = 7, *n* = 4, respectively. Error bars indicate standard errors. *, ** indicate significant differences. (Student’s *t*-test: *p < 0.05, **p < 0.01)

Since we confirmed that strain J8 is capable of nodulation on *G. glabra*, we next investigated the effect of strain J8 inoculation on the plant growth at 30 DAI. No significant difference was detected between J8-inoculated and uninoculated plants in shoot length, shoot weight, root length and root weight (Fig. 2A–D). On the other hand, SPAD values, a measure of leaf chlorophyll content, were significantly higher when inoculated with strain J8 (Fig. 2E). Furthermore, when the number of nodules and nitrogen-fixing activity were examined, only a few nodules were formed in the uninoculated plants, whereas an overwhelmingly large number of nodules were formed in the J8-inoculated plants (Fig. 2F, G). Acetylene reduction activity (ARA), an indicator of nitrogen-fixing activity, was significantly higher in the case of strain J8 inoculation than that of uninoculation (Fig. 2H). These results suggest that inoculation with the strain J8 resulted in efficient nodulation, which supplied more nitrogen to the leaves, thereby increasing the nitrogen content in the leaves [20].

### Effect of strain J8 on GL synthesis marker genes expression at 30 DAI

**Fig. 3:**
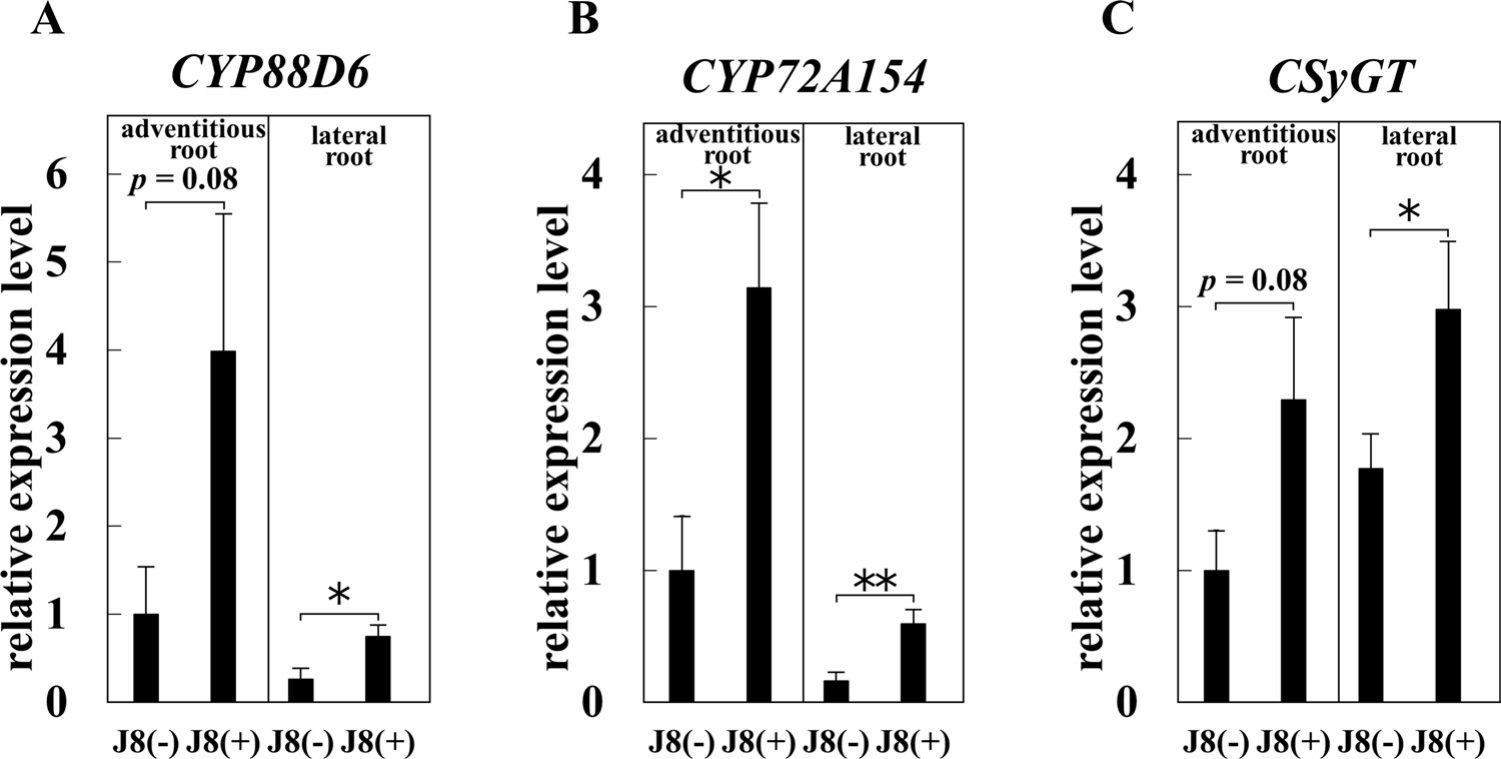
Expression of genes involved in the GL biosynthetic pathway in the root of *G. glabra* at 30 days after rhizobium inoculation. (*n* = 4 in uninoculated lateral root or J8-inoculated adventitious root, *n* = 5 in uninoculated adventitious root or J8-inoculated lateral root) Expression levels of *CYP88D*6 gene (**A**), *CYP72A154* gene (**B**), *CSyGT* gene (**C**). *18S rRNA* gene was used as the internal control. For the respective gene, the expression level of J8(−) in the adventitious root was set to 1. Error bars indicate standard errors. *, ** indicate significant differences. (Student’s *t*-test: *p < 0.05, **p < 0.01)

We investigated the effect of rhizobial inoculation on GL production at 30 DAI. However, attempts were failure due to the values below detection limit. Therefore, we investigated the expression of *CYP88D6*, a GL synthesis marker gene that has been shown to have a strong positive correlation with GL production in previous studies [21], and *CYP72A154* and *CSyGT*, two genes involved in the process from* β*-amylin to GL [22, 23]. In this experiment, the adventitious roots arising from the base and lateral roots arising from adventitious roots were divided, and their expressions were analyzed. In the adventitious roots, the expression of *CYP88D6* and *CSyGT* tended to increase in J8-inoculated plants (Fig. 3A, C), and *CYP72A154* was significantly up-regulated by strain J8 inoculation (Fig. 3B).The expression analysis using lateral roots showed that all three genes were significantly up-regulated in strain J8-inoculated plants (Fig. 3). These results suggested that GL production in *G. glabra* is enhanced by rhizobial inoculation.

### Effect of strain J8 on *G. glabra* growth at 150 DAI

**Fig. 4:**
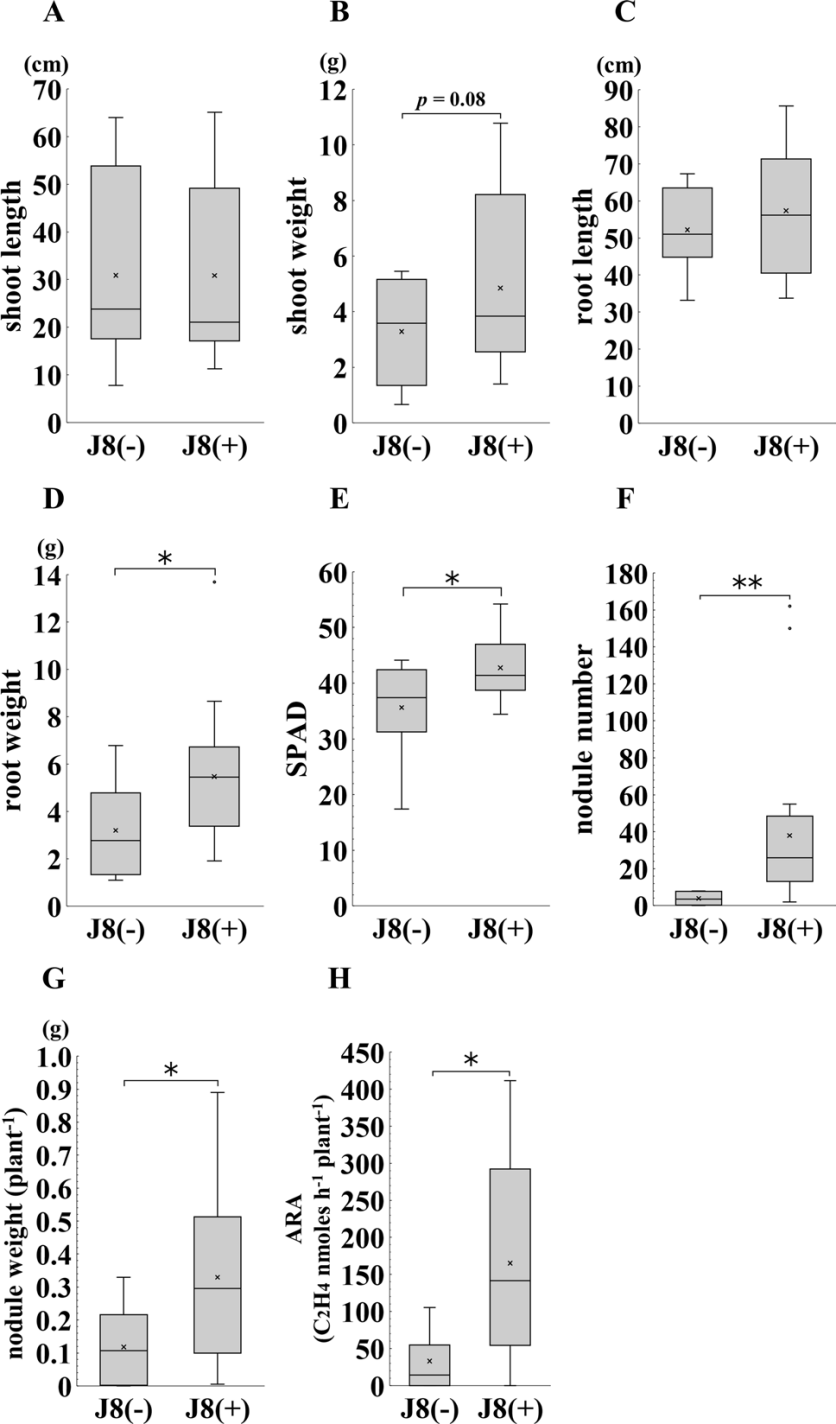
Growth, nodulation and nitrogen fixation activity of *G. glabra* prepared from cuttings grown at 150 days after rhizobium inoculation. Shoot length (**A**), shoot weight (**B**), root length (**C**), root weight (**D**), leaf SPAD value (**E**), number of nodules (**F**), nodule weight (**G**), acetylene reduction activity (**H**) was measured. Shoot length, shoot weight, root weight, SPAD value, nodule number, and nodule weight; J8(−), J8(+), *n* = 8, *n* = 21, respectively. Root length; J8(−), J8(+): *n* = 8, *n* = 16, respectively. Acetylene reduction activity; J8(−), J8(+): *n* = 7, *n* = 17, respectively. Error bars indicate standard errors. *, ** indicate significant differences. (Student’s *t*-test: *p < 0.05, **p < 0.01)

**Fig. 5:**
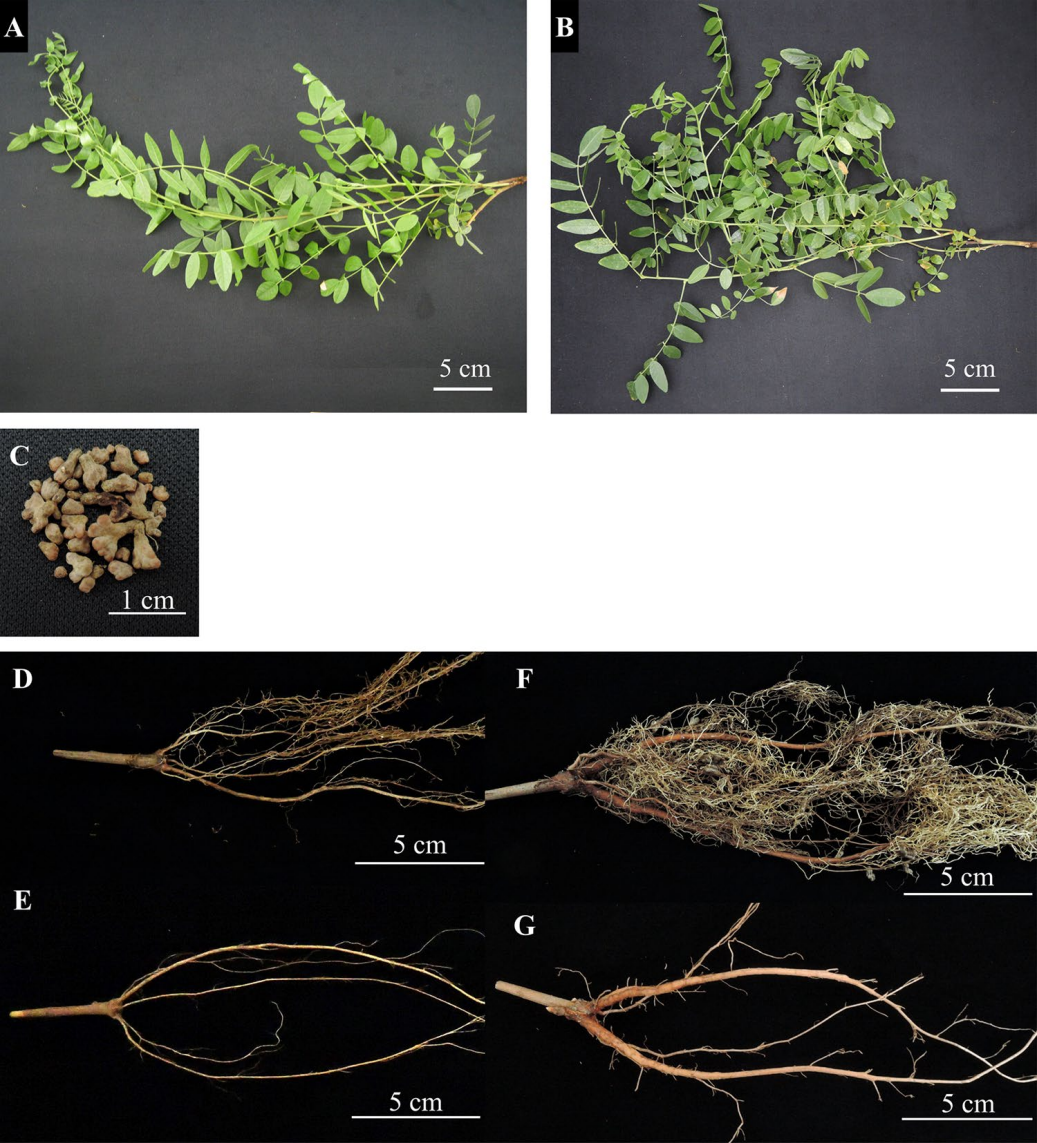
Root phenotype of *G. glabra* prepared from cuttings and grown for 150 days after rhizobial inoculation. Phenotypes of shoot of uninoculated plants (**A**), shoot of plants inoculated with rhizobia (**B**), nodules formed on roots (**C**), roots of uninoculated plants (**D**), thick roots with thin roots removed from uninoculated roots (**E**), roots of plants inoculated with rhizobia (**F**), thick roots with fine roots removed from roots of plants inoculated with rhizobia (**G**)

When strain J8 was inoculated and allowed to grow for 30 days, it was suggested that a large amount of nitrogen, which increase nitrogen content of plant leaves was provided through rhizobial symbiosis. As shown in Fig. 2A–E, although an increase in SPAD value was observed, no effect on biomass production was detected. Therefore, we extended the growth period to 150 days and investigated the effect on the growth of host plant (Figs. 4, 5). No significant difference was detected in shoot length and root length (Fig. 4A, C), but significant increase or increasing trend were observed in shoot weight and root weight (Fig. 4B, D). SPAD value was significantly higher by strain J8 inoculation as well as the case of 30 days (Figs. 4E, 5A, B). Next, the number of nodules and nitrogen-fixing activity were investigated 150 DAI. The results showed that a few nodules were formed without inoculation as well as the case of 30 days, but more nodules were formed in the J8-inoculated plants (Figs. 4F, 5C). Also, the total nodules weight per plant was significantly heavier in the case of strain J8 inoculation (Fig. 4G). The nitrogen-fixing activity of J8-inoculated plants was significantly higher than that of uninoculated plants (Fig. 4H).

**Fig. 6:**
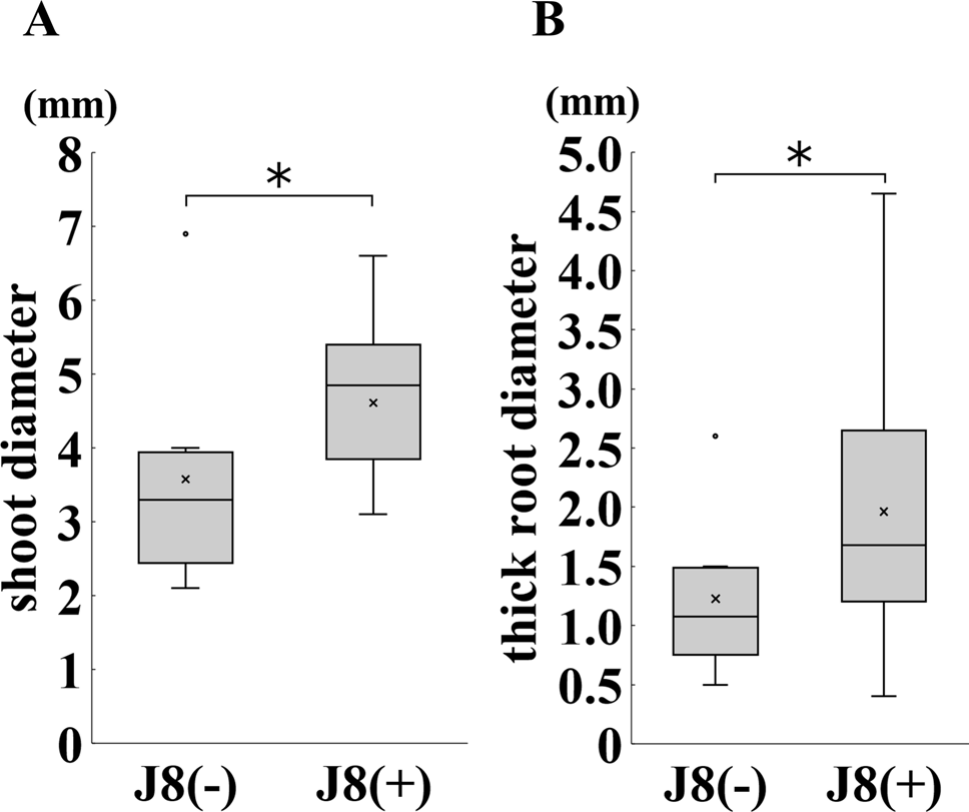
Shoot and root diameters of *G. glabra* prepared from cuttings grown for 150 days after rhizobial inoculation. Shoot and thick root diameters were measured at 1 cm above and below the base. J8(−), J8(+): *n* = 8, *n* = 19, respectively. Shoot diameter (**A**), root diameter (**B**) was measured. Error bars indicate standard errors. *, ** indicate significant differences. (Student’s *t*-test: *p < 0.05, **p < 0.01)

Next, since it was expected that the size of diameter of the shoots and roots around the base would differ between rhizobium inoculated and uninoculated plants (Fig. 5D–G), the diameters of the shoots and roots were measured. Figure 6A, B showed that both shoots and roots became thicker after inoculation with rhizobium, confirming that the growth were promoted.

These results indicate that rhizobial inoculation promotes plant growth at 150 DAI. Especially in the case of *Glycyrrhiza*, where the thick roots are dried and used as a product, the enlargement of roots is a so advantageous property.

### Effect of strain J8 on GL synthesis marker genes expression and GL production at 150 DAI

**Fig. 7:**
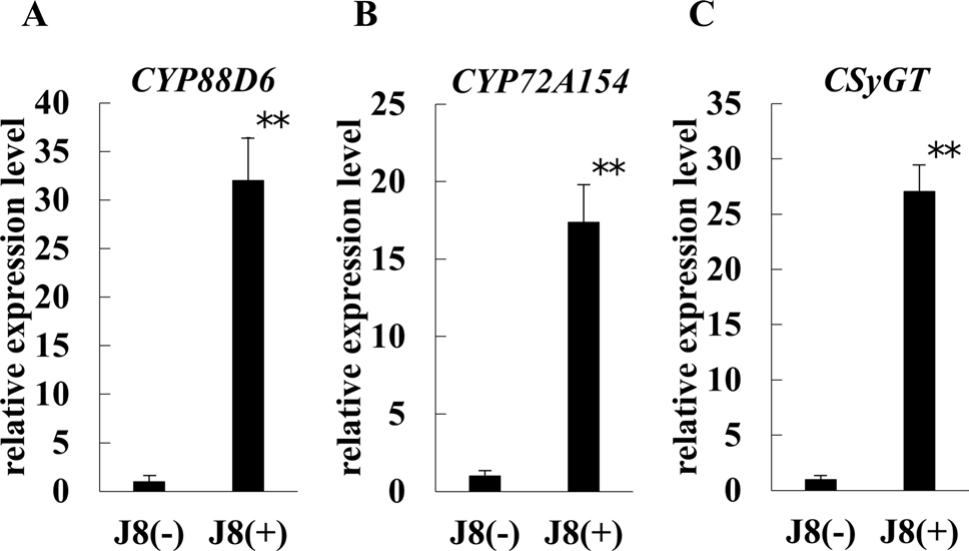
Expression of genes involved in the GL biosynthetic pathway in the root of *G. glabra* at 150 days after rhizobial inoculation. Expression levels of *CYP88D6* gene (**A**), *CYP72 A154* gene (**B**), *CSyGT* gene (C). J8(−), J8(+): *n* = 5, *n* = 5. *18S rRNA* gene was used as the internal control. For the respective gene, the expression level of J8(−) was set to 1. Error bars indicate standard errors. *, ** indicate significant differences. (Student’s *t*-test: *p < 0.05, **p < 0.01)

**Fig. 8:**
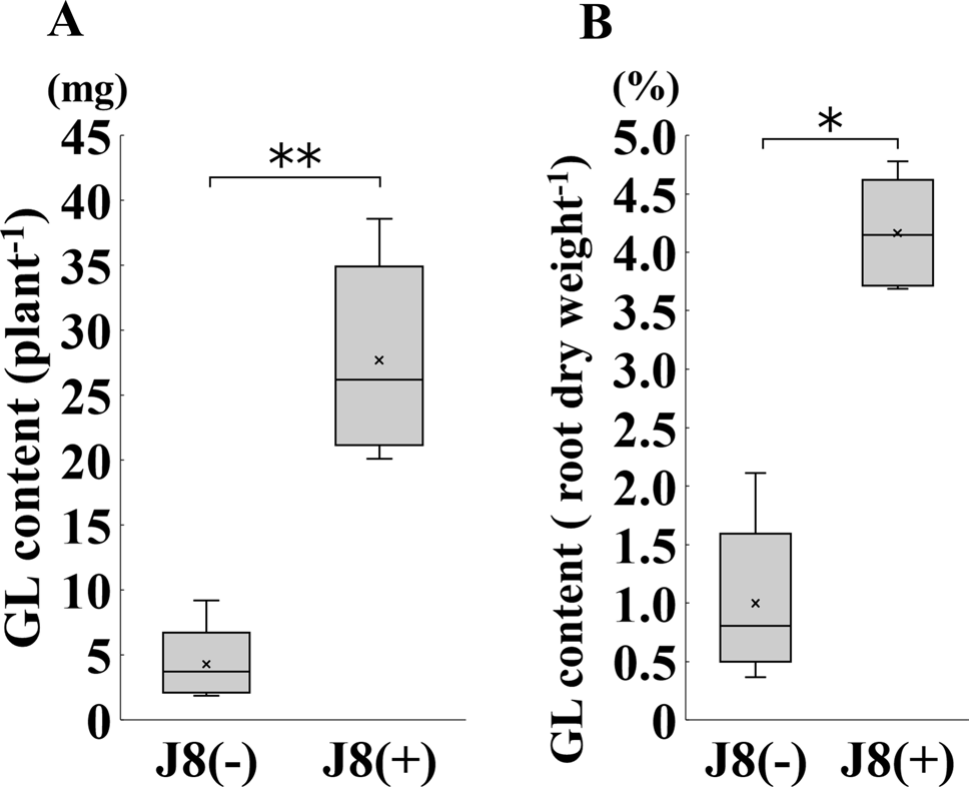
GL content of *G. glabra* prepared from cuttings and grown for 150 days after rhizobium inoculation. Total GL content per unit of roots weight (**A**), GL content per root dry weight (**B**) was measured. J8(−), J8(+); *n* = 5. Error bars indicate standard errors. *, ** indicate significant differences. (Student’s *t*-test: *p < 0.05, **p < 0.01)

Expression analysis of three genes, *CYP88D6*, *CYP72A154*, and *CSyGT*, were also performed at 150 DAI. As shown in Fig. 7, the expression of *CYP88D6* gene, *CYP72A154* gene, and *CSyGT* gene increased significantly by about 32 times, 17 times, and 27 times, respectively, after inoculation with strain J8 compared to uninoculation. Next, the effect of strain J8 inoculation on GL production after 150 days was investigated. *Glycyrrhiza* is generally considered to have a high GL content in the main roots and stolons. Therefore, the GL content was measured only the thick root with a diameter of at least 2 mm at 1 cm from the base. In the case of uninoculation, the entire root was used as a sample because no thick roots larger than 2 mm were present. As a result, the GL content per plant was significantly increased by about 6 times in the thick roots compared to the uninoculated roots, as shown in Fig. 8A. Moreover, as shown in Fig. 8B, the GL content in only thick roots was significantly increased to about 4% with strain J8 was inoculated.

### Involvement of JA in the mechanism of increased GL production by rhizobial inoculation

**Fig. 9:**
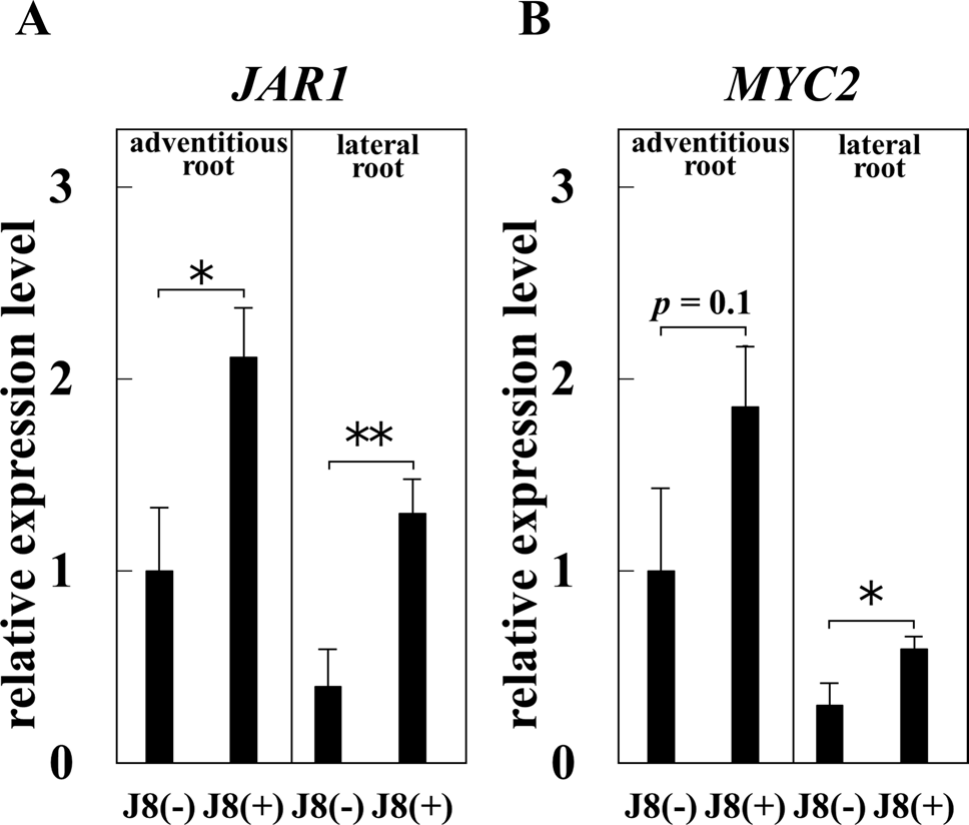
Expression of JA synthesis marker genes in the root of *G. glabra* at 30 days after rhizobial inoculation (*n* = 4 in uninoculated lateral root or J8-inoculated adventitious root, *n* = 5 in uninoculated adventitious root or J8-inoculated lateral root) Expression levels of *JAR1* gene (**A**), *MYC2* gene (**B**). *18S rRNA* gene was used as the internal control. For the respective gene, the expression level of J8(−) in the adventitious root was set to 1. Error bars indicate standard errors. *, ** indicate significant differences. (Student’s *t*-test: *p < 0.05, **p < 0.01)

**Fig. 10:**
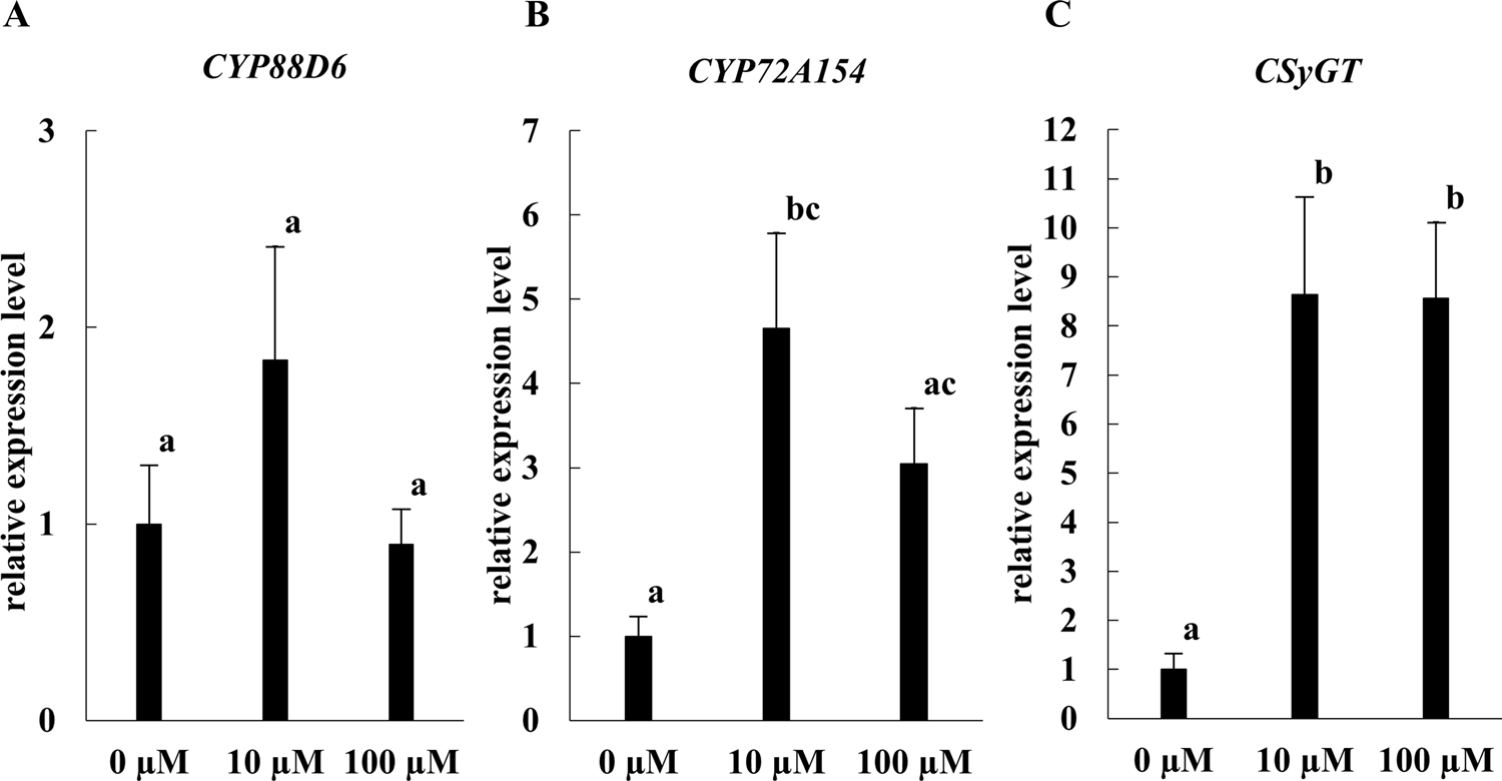
Expression of genes involved in the GL biosynthetic pathway in the root of *G. glabra* on MeJA-treated. Expression levels of *CYP88D6* gene (**A**), *CYP72A154* gene (**B**), and *CSyGT* gene (**C**). (0 µM: *n* = 5, 10 µM: *n* = 5, 100 µM: *n* = 5) *18S rRNA* gene was used as the internal control. For the respective gene, the expression level of J8(−) in the adventitious root was set to 1. Error bars indicate standard errors. Different letters indicate significant differences (*p* < 0.05). Statistical methods were performed using analysis of variance (ANOVA) and the Turkey method

Although strain J8 inoculation promoted plant growth and GL production on *G. glabra*, the mechanism by which rhizobial inoculation increased GL production is unknown. GL is a triterpenoid saponin, and it has been reported that the biosynthesis of triterpenoid saponins and terpenoid compounds is enhanced by MeJA and JA [24]. It has also been reported that JA biosynthesis is enhanced in *G. max* by inoculation with rhizobium [25]. Therefore, we developed a working hypothesis that strain J8 inoculation promotes JA synthesis in *Glycyrrhiza*, which in turn promotes GL production. First, we investigated whether strain J8 inoculation affected the expression of JA synthesis marker genes in roots. As shown in Fig. 9, *JAR1* and *MYC2* gene expression was significantly up-regulated in inoculated lateral and adventitious root. Next, we tested whether the expression of the three GL synthesis marker genes was responsive to JA. Because MeJA treatments on *G. uralensis* were performed at 100 µM MeJA in previous study [8], we analyzed the expression of the three genes, *CYP88D6*, *CYP72A154*, and *CSyGT* using *G. glabra* treated with 10 or 100 µM MeJA. The results showed that the expression of *CYP72A154* and *CSyGT* were significantly up-regulated compared to 0 µM treatment (Fig. 10). These results suggested that JA production was enhanced by rhizobial inoculation, thereby leading to increased expression of GL synthesis marker genes.

## Discussion

In this study, we confirmed that the strain J8 efficiently formed nodules and fixed nitrogen on the root of *G. glabra*. In a previous study, when isolated rhizobia were re-inoculated on *G. glabra* without KNO_3_, no nodules formed. Nitrate nitrogen is known to be one of the factors involved in the control of nodulation [18, 19]. Therefore, we investigated whether nodules form in a medium with 1 mM KNO_3_. As a result, an overwhelming number of nodules were formed in the nitrogen-added medium (Figs. 1, 2F). These results suggested that the presence of nitrogen is an advantageous factor for establishment of symbiosis between *G. glabra* and strain J8. In our experimental condition, a few nodules were formed on the uninoculated root. This nodulation may have been due to contamination by airborne rhizobia. Other studies have reported that symbiosis with mycorrhizal fungi promotes root growth and GL production in *G. glabra* [26]. In this study, airborne rhizobium bacteria were contaminated and formed nodules on uninoculated plants. However, when the roots were observed with trypan blue staining, the, presence of mycorrhizal fungi was not overserved. Therefore, it is unlikely that the enhancement of root growth and GL production was due to the infection of mycorrhizal fungi.

Although biomass production did not always increased 30 DAI, but also SPAD values of inoculated plants were significantly higher than those of uninoculated plants (Fig. 2E). Since SPAD values are reported to be positively correlated with the nitrogen content of plants [18], more nitrogen may have been supplied by rhizobial symbiosis in inoculated plants. Then, at the stage 150 DAI, biomass production and SPAD value significantly increased compared with uninoculated plant (Figs. 4D, E, 6B). These results suggested that it took more than 30 days for the rhizobial symbiosis to positively affect the growth of *G. glabra* under the conditions used this experiment. We observed that the root become hyper-trophied at 150 days after rhizobial inoculation (Fig. 6B). Since the thick root are generally used as the product, root enlargement is an exceedingly advantageous property in *Glycyrrhiza*. However, the cause of root enlargement by inoculation with rhizobium is unknown. In rice, plant growth-promoting rhizobacteria such as *Bacillus* sp. have been reported to promote root growth [27]. Since the promotion of root growth by *Bacillus* sp. has been attributed to the plant’s defense response to *Bacillus* sp. [28], it is possible that a similar response may occur in the case of rhizobia. Therefore, the effect of rhizobial inoculation on the physiological activity of *Glycyrrhiza* should be investigated in the future.

Inoculation with strain J8 was increased the expression levels of GL biosynthesis related genes such as *CYP88D6*, *CYP72A154*, and *CSyGT* (Figs. 3, 7). *Glycyrrhiza* contains a large amount of GL in its main roots and stolons. Therefore, the roots were divided into adventitious and lateral roots, and the changes in expression were analyzed at 30 days after rhizobial inoculation. The results showed that the expression of these genes was significantly increased or tended to increase in adventitious and lateral roots. Although GL production could not be confirmed by HPLC at 30 days after rhizobial inoculation, the expression of three GL synthesis marker genes was increased or trended to increase in both adventitious and lateral roots, suggesting that GL production was already active. Since the thick root of *Glycyrrhiza* is used as a crude drug, a thick root from which thin roots have been removed. We defined root lager than 2 mm as thick root in plant inoculated with rhizobium at 150 days and analyzed gene expression. In the case of uninoculated plants, whole roots were used as samples because no roots larger than 2 mm were present. As a result, the three GL synthesis marker genes were dramatically up-regulated by rhizobial inoculation even after 150 days (Fig. 7). This was thought to reflect the overwhelming increase in GL production. At 30 DAI, expression levels of *CYP88D6*, *CYP72A154*, and *CSyGT* increased approximately 4 times, 2 times, and 3 times, respectively, with rhizobial inoculation, while at 150 DAI they increased approximately 32 times, 17 times, and 27 times, respectively. This result suggested that the increase in GL production due to rhizobial inoculation has a greater effect at 150 DAI than at 30 DAI. Because GL is a specialized metabolite, it is actively produced when plants trigger defense response to biotic, environmental, or other stresses. Since rhizobial infection is a kind of biotic stress for the host plant, the expression of genes involved in the GL biosynthetic pathway may have been increased by rhizobial inoculation. Therefore, in the future, it is necessary to as certainly how the expression of these genes changes with the number of days after rhizobial inoculation.

GL content increased to more than 4% in the thick root at 150 days after rhizobial inoculation (Fig. 8B). Under the current cultivation method of *Glycyrrhiza* commonly used in Japan, it takes more than three years of growth to meet the standard for GL content. In this study, only 150 DAI with strain J8 the GL content was found to exceed the standard. These results indicated that the trade-off between biomass production and GL production in *Glycyrrhiza* could be resolved by rhizobial symbiosis with strain J8. On the other hand, in our previous report, the GL content of *G. uralensis* at 90 DAI was about 0.2% when inoculated with rhizobia [13], whereas in this study, GL content exceeded 4% at 150 DAI in *G. glabra.* The promotion of GL production by rhizobial inoculation seems to differ greatly among the different *Glycyrrhiza* species, but direct comparisons cannot be made because of the different growth stages. However, since the same rhizobium were used in *G. uralensis* and *G. glabra*, there is still a possibility that the host and rhizobium are compatible with each other. Therefore, future comparisons should be made at the same growth stage.

We analyzed the expression of JA synthesis marker genes to elucidate the mechanism by which J8 inoculation increased GL production. As a result, the expression of *JAR1* and *MYC2*, JA synthesis marker genes, increased at 30 days after J8 inoculation (Fig. 9). Next, we investigated whether three genes involved in GL biosynthesis responded to MeJA, and the expression of *CYP72A154*, and *CSyGT* were significantly increased by MeJA treatment (Fig. 10). These results suggest a scheme in which JA production is increased by rhizobial inoculation, and GL production is increased by a response of GL marker genes. This scheme was consistent with the working hypothesis we had established. However, no response of the *CYP88D6* gene to MeJA was observed. Various phytohormones have been reported to be involved in rhizobial symbiosis [29, 30]. In the future, the effects of phytohormones involved in symbiosis on the expression of *CYP88D6* should be investigated. The details of the mechanism by which inoculation with the J8 strain increases GL production are unknown. To elucidate the mechanism of increased GL production by inoculation with strain J8, it is necessary in the future to comprehensively analyzed the change in physiological activity of the plant by performing transcriptome analysis at different growth stages after inoculation with strain J8. In addition to GL, other metabolites such as liquiritin are also produced in *G. glabra* [31]. Liquiritin and isoliquiritin are known flavonoid compounds, and it has been reported that flavonoid compounds are important in the early stages of legume-rhizobium symbiosis. Therefore, it is possible that when *G. glabra* establishes rhizobial symbiosis, the production of metabolites other than GL may also increase. Future work should include metabolomic analysis of *G. glabra* to determine how rhizobial inoculation alters the production of all metabolites.

Moreover, in order to popularize the cultivation of *Glycyrrhiza* using rhizobia in society, it is necessary to establish a more practical cultivation method.
